# Bidirectional Relationship Between Insomnia and Depressive Symptoms in Family Caregivers of People with Dementia: A Longitudinal Study †

**DOI:** 10.3390/bs15070936

**Published:** 2025-07-10

**Authors:** Lucía Jiménez-Gonzalo, María Márquez-González, Carlos Vara-García, Rosa Romero-Moreno, Javier Olazarán, Roland von Känel, Brent T. Mausbach, Andrés Losada-Baltar

**Affiliations:** 1 Department of Psychology, Universidad Rey Juan Carlos, 28922 Madrid, Spain; carlos.vara@urjc.es (C.V.-G.); rosa.romero@urjc.es (R.R.-M.); andres.losada@urjc.es (A.L.-B.); 2 Department of Biological and Health Psychology, Universidad Autónoma de Madrid, 28049 Madrid, Spain; maria.marquez@uam.es; 3 Department of Neurology, Hospital General Universitario Gergorio Marañon, 28007 Madrid, Spain; javier@mariawolff.es; 4 Department of Consultation-Liaison Psychiatry and Psychosomatic Medicine, University Hospital Zürich, University of Zürich, 8032 Zürich, Switzerland; roland.vonkaenel@usz.ch; 5 Department of Psychiatry, University of California San Diego, La Jolla, CA 92093, USA; bmausbach@health.ucsd.edu

**Keywords:** caregiving, stress, distress, sleep, mixed models

## Abstract

Bidirectionality between insomnia and depression is well documented in general and clinical populations but remains under-researched in family caregivers of people with dementia. This study aimed to explore this relationship using a longitudinal design with 155 family caregivers assessed annually over three years. Data collected included sociodemographic information, health behaviors, medical data, caregiving stressors, and depressive and insomnia symptoms. Two linear mixed models were tested: Model 1 considered insomnia symptoms as the independent variable and depressive symptoms as the outcome; Model 2 considered depressive symptoms as the independent variable and insomnia symptoms as the outcome. The results showed that caregivers with more insomnia symptoms over time had significantly higher depressive symptoms, even after adjusting for covariates. Insomnia accounted for an additional 7.47% of the variance, with a total explained variance of 57.93%. Conversely, higher depressive symptoms over time were associated with increased insomnia. Depressive symptoms explained an additional 7.28% of the variance, with a total explained variance of 25.74%. These results were consistent with previous studies on non-caregiving populations, adding empirical evidence to the notion that both insomnia and depression may operate as a risk factor for the other disorder. Caregiver support interventions could improve their psychological well-being if they incorporate sleep-focused strategies.

## 1. Introduction

Most people living with dementia are cared for by family members (Schulz et al., 2020). Family caregivers of people with dementia often identify positive aspects of care, such as love or reciprocity, personal growth, sense of meaning, or gratitude (Brodaty & Donkin, 2009; Quinn & Toms, 2019). Yet, the caregiving experience is usually seen as a highly stressful situation that may last up to several years (Schulz et al., 2020) and that has been associated with negative consequences for caregivers’ physical and mental health (Knight & Sayegh, 2010; Schulz et al., 2020), including insomnia (Brewster et al., 2022; Gao et al., 2019) and depression (Collins & Kishita, 2020). Notably, 50–74% of family caregivers of people with dementia report sleep problems (Ma et al., 2018), and 31.2% of caregivers present clinically significant depressive symptoms, according to a meta-analysis by Collins and Kishita (2020). These rates are higher than the ones reported in non-caregivers (Ma et al., 2018). Most research on this topic has been done under theoretical frameworks that highlight the stressful nature of caring for a relative with dementia (Knight & Sayegh, 2010; Vitaliano et al., 2004), with the behavioral and psychological symptoms of dementia (BPSD) being a main source of stress (Ma et al., 2018).

Although the problem of bidirectionality between symptoms of insomnia and depression has been recognized in this population (Vitaliano et al., 2004), this issue has not been adequately researched. Most research analyzing the consequences of caregiving for caregivers’ depression and insomnia have used a cross-sectional design and analyzed the consequences in a unidirectional way, not considering the potential interrelation between these outcomes. Several cross-sectional studies done with family dementia caregivers start from a rationale in which depression is assumed to be a predictor of the onset of insomnia (McCurry et al., 2015; Peng et al., 2018). A recent systematic review (Brewster et al., 2022) concluded that caregivers’ depressive symptoms may be a variable that triggers insomnia.

However, other studies started from the hypothesis that insomnia symptoms are the predictor of the onset of depression in the caregiving population. For example, Wang et al. (2016) found that caregivers’ sleep quality mediates the relationship between the distress caused by care-recipients’ BPSD and caregivers’ depressive symptoms. Honda et al. (2017) found caregivers’ sleep quality to mediate the association between care-recipients’ BPSD and caregivers’ psychological distress. McCurry et al. (2015) suggested that caregiver depression is a risk factor for the onset of new sleep disturbances but acknowledged sleep problems to be themselves a risk factor for the onset of depression. Thus, caregivers may develop a self-perpetuating cycle of sleep and mood disturbances that is difficult to break (McCurry et al., 2015). However, to the best of our knowledge, this hypothesis has not been empirically tested yet in family dementia caregivers.

This bidirectional relationship is well documented in clinical populations. In participants with clinical depression, insomnia symptoms have traditionally been considered a secondary manifestation of depression (Fang et al., 2019; Freeman et al., 2020). However, this perspective has been challenged by a range of studies (e.g., Alvaro et al., 2013; Franzen & Buysse, 2008; Sivertsen et al., 2012). A longitudinal study by Sivertsen et al. (2012) identified insomnia as an independent risk factor for the development of emerging or recurrent depression among adults and also found depression to be an independent risk factor for the development of insomnia. Other longitudinal studies (Rothe et al., 2020) found both insomnia and depression to significantly predict the onset of the other disorder. Review papers on longitudinal, epidemiological, and intervention studies (Alvaro et al., 2013; Freeman et al., 2020) also identified this bidirectional association between insomnia and depression. However, Alvaro et al. (2013) claimed that several of the reviewed studies did not control for important potential covariates and recommended future research to account for variables such as drug and alcohol use and exercise.

Thus, longitudinal and experimental designs are needed to further study the causal relationship in the dementia caregiving population. In a systematic review by Peng and Chang (2013), 17 of the 18 studies considered used a cross-sectional design. Similarly, Brewster et al. (2022) only found three studies with a longitudinal design that included the measurement of sleep in family caregivers (von Känel et al., 2012, 2014; McCurry et al., 2008). This limits our understanding of how depression and insomnia evolve during the caregiving process. Considering that the prevalences of depression (Collins & Kishita, 2020) and insomnia (Ma et al., 2018) in caregivers are clearly higher than the ones observed in the general population, it is unclear if the bidirectional association observed in the general population will also be observed in caregivers, or if one of the studied variables will have a major role in the longitudinal association between variables. Thus, the longitudinal approach helps to provide a clearer picture of the development, interplay, and evolution between symptoms of depression and insomnia in family dementia caregivers (Peng & Chang, 2013). The aim of the present study is to address two gaps in the current literature in family dementia caregivers: first, to analyze the time-varying associations between depression and insomnia symptoms with a longitudinal approach and data analyses via mixed models; and second, to empirically test a longitudinal model that considers a broad range of potential covariates in the dementia caregiving stress process. Drawing upon previous studies (Rothe et al., 2020; Sivertsen et al., 2012) and Vitaliano’s model of caregiving stress and health (2004), our research hypotheses were as follow:

**H1.**  *Insomnia symptoms will be longitudinally associated with caregivers’ depressive symptoms after controlling for sociodemographic, health behaviors, medical issues, and caregiving stressors.*

**H2.**  *Depressive symptoms will be longitudinally associated with caregivers’ insomnia symptoms after controlling for sociodemographic, health behaviors, medical issues, and caregiving stressors.*

## 2. Materials and Methods

### 2.1. Participants

One hundred and fifty-five caregivers of a relative with dementia living in the community in Madrid (Spain) took part in this study. The inclusion criteria were (1) to identify oneself as the primary caregiver of a relative with dementia, (2) to devote at least one hour per day to caregiving tasks, (3) to provide care for at least three consecutive months, and (4) to be at least 18 years old. The exclusion criteria were: (1) providing care for less than one hour per day or seven hours per week, (2) assuming the caregiving role less than 3 months ago, (3) attending weekly individual psychological or psychiatric counselling, and (4) cared-for family member not having mild cognitive decline or dementia.

### 2.2. Procedure

Participants were recruited through medical, social, and daycare centers in Madrid. To verify if caregivers met the inclusion criteria, participants were initially contacted via telephone. Then, face-to-face interviews were conducted by a trained clinical psychologist at the different centers involved in the study. Since May 2020, due to the COVID-19 pandemic-related sanitary and social distancing measures, interviews were telephone-based. Over the course of this study, three yearly assessments were conducted: at baseline, at 1-year follow-up, and at 2-years follow-up. Participation in the study was not terminated if the care recipient transited to a long-term care facility or deceased. All participants gave informed consent to participate in the study, which was approved by the Ethics Committee from Rey Juan Carlos University (Madrid, Spain) (register number: 0210202017620).

**Figure 1:**
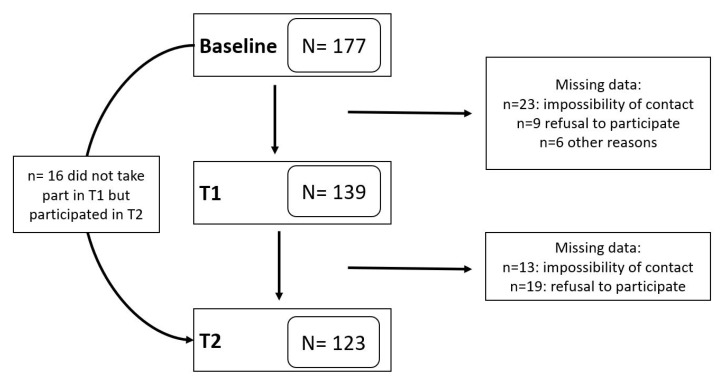
Flowchart of the study.

Three hundred and fifty-eight caregivers were initially contacted via telephone. Of these, 54 did not take part in the study because they did not meet the inclusion criteria, 21 participants could not be contacted after the first phone call, and 106 declined to participate. A flowchart of the participants included in the study can be seen in Figure 1. Of the 177 initial participants, 22 did not respond to at least two time points, so they were not considered for the final analysis.

### 2.3. Variables and Instruments

#### 2.3.1. Sociodemographic Information

Information on gender, age, months of caregiving, and daily hours devoted to caregiving tasks was collected. At baseline assessment, the variable “months caregiving” reflected how long the caregivers had carried out this role before entering the study. Caregiving cessation was assessed by asking the caregiver “Are you still the main caregiver of your relative?”, and answers were coded as 0 = “still the main caregiver”, 1 = “no longer the main caregiver”.

#### 2.3.2. Health Behaviors

Data were collected regarding weekly hours of exercise, average alcohol intake during the last month, and smoking status (coded as 0 = “non-smoker”, 1 = “smoker”).

Frequency of leisure activities was assessed using an adaptation of the Leisure Time Satisfaction Scale (Stevens et al., 2004). This six-item scale measured caregivers’ frequency of engagement in six different pleasant events over the past month (e.g., “How often have you participated in hobbies or other interests?”). Answers were 0 = “not at all”; 1 = “a bit”; 2 = “a lot”. For the present study, Cronbach’s alpha was 0.71.

#### 2.3.3. Medical Data

Caregivers were asked “Have you been diagnosed with any of the following health problems?” and given a list with 17 items (e.g., arthritis, heart disease, diabetes). The number of health problems were aggregated.

Caregivers were asked about their prescribed medications. Then, the number of anxiolytics (e.g., lorazepam), antidepressants (e.g., sertraline), and hypnotic medication (e.g., zolpidem) the caregiver reported taking during the previous year was added.

#### 2.3.4. Caregiver Stressors

Behavioral and Psychological Symptoms of Dementia (BPSD). The revised memory and behavior problems checklist (RMBPC) (Teri et al., 1992) was used. This 24-item scale assesses the emotional distress in the caregiver caused by BSD (e.g., repeating the same question), with responses ranging from 0 = “not at all distressed” to 4 = “extremely distressed”. The scale was adapted to the Spanish population by Nogales-González et al. (2015). Cronbach’s alpha for this study was 0.87.

#### 2.3.5. Depressive Symptoms

Depressive symptomatology was assessed using the Center for Epidemiologic Studies-Depression Scale (CES-D) (Radloff, 1977), adapted for Spanish caregivers by Losada-Baltar et al. (2012). The scale consists of 20 items assessing depressive symptoms during the previous week, with responses ranging from 0 = “rarely or never” to 3 = “most or all of the time”. A cutoff score of 16 was used to identify participants with clinically relevant depressive symptoms (Lewinsohn et al., 1997). Cronbach’s alpha for this study was 0.90.

#### 2.3.6. Insomnia Symptoms

Caregivers’ insomnia symptoms were assessed using the Insomnia Severity Index (ISI) (Morin, 1993). This seven-item scale measures subjective insomnia symptoms, such as difficulty falling asleep. The ISI has been validated for its use with family dementia caregivers (Jiménez-Gonzalo et al., 2021). A cutoff score of 10 was applied to identify participants with clinically relevant insomnia symptoms (Morin et al., 2011). Cronbach’s alpha for this study was 0.83.

### 2.4. Data Analysis

Data were analyzed using the Statistical Program for Social Sciences (SPSS version 29.0). To examine the bidirectional association of insomnia and depressive symptoms over time, linear mixed models were used, with the restricted maximum likelihood (REML) method being implemented to handle missing data. Mixed model regression could be used to estimate an intercept and slope for each participant based on the available data for that individual (i.e., even when some points are missing across assessments), augmented by the data from the entire sample (Singer & Willett, 2003). Effect sizes are expressed as pseudo-R^2^, which indicates the amount of variance of the outcome that is explained by a model’s specific combination of independent variables. The overall fit of the model was tested using Akaikie’s Information Criteria (AIC), with smaller values indicating a better model fit.

Covariates were selected a priori based on previous research showing that these may affect caregivers’ depression and insomnia (Alvaro et al., 2013; McCurry et al., 2015; von Känel et al., 2012). Thus, the model included the following fixed effects: age at baseline, sex (dummy coded as 0 = “male”, 1 = “female”), months of caregiving, daily hours of care, health problems, caregiving cessation (dummy coded as 0 = “still the main caregiver”, 1 = “no longer the main caregiver”), weekly hours of exercise, leisure activities, alcohol intake per month, smoking status (dummy coded as 0 = “non-smoker”, 1 = “smoker”), medication intake, and distress due to BPSD. The bidirectional relationship between symptoms of depression and insomnia was tested in two ways. In Model 1, insomnia symptoms were entered as the independent variable and depressive symptoms as the outcome variable. In Model 2, depressive symptoms were entered as the independent variable and insomnia symptoms as the dependent variable. All variables except for age at baseline and sex were entered as time varying. Random intercepts were modeled for participants. To increase the interpretability of the regression coefficients and reduce problems associated with multicollinearity, linear variables were centered at their grand means. Since linear independent variables were centered at their grand means, the intercept represents mean depressive symptoms (Model 1) or mean insomnia symptoms (Model 2) scores at baseline for male caregivers with no caregiving cessation, with average scores in the assessed independent variables.

Secondary analyses were carried out to explore whether time spent as a caregiver was associated with depression and insomnia symptoms. Spearman correlation coefficient was calculated between time caregiving and depression or insomnia symptoms to test for linear associations. Also, one-way ANOVAs were used to test whether there were differences in depression and insomnia mean scores depending on the amount of time that caregivers were performing this role at baseline.

The present study was not preregistered. The data presented in this study are available on request from the corresponding author due to the ongoing status of the project.

## 3. Results

### 3.1. Characteristics of the Sample

Table 1 shows the characteristics of study participants at baseline and for the subsequent yearly assessments. Of the 155 caregivers assessed at baseline, data were available for 139 (89.68%) at 1-year follow-up, and for 122 caregivers (78.70%) at 2-year follow-up. At baseline, about one-fourth of caregivers had clinically relevant insomnia symptoms (ISI scores ≥ 10) and more than one-third had clinically relevant depressive symptoms (CES-D scores ≥ 16). The results also showed that caregivers took a maximum of three different psychiatric medications on a regular basis (i.e., anxiolytics, antidepressants, or hypnotics).

### 3.2. Model 1: Longitudinal Relationship Between Insomnia and Depressive Symptoms

The results of the linear mixed model analyzing the relationship between insomnia and depressive symptoms after controlling for covariates are shown in Table 2. The coefficient for “time” was not significant, indicating that caregivers showed no significant changes in depressive symptoms over time. Table 2 shows that caregivers with more insomnia symptoms over the study period had significantly higher depressive symptoms also over the study period (β = 0.41; *p* < 0.001). This association remained significant after controlling for covariates. Additionally, younger age (β = −0.15; *p* < 0.01), fewer leisure activities (β = −0.94; *p* < 0.001), more medication intake (β = 2.31; *p* < 0.01), and distress caused by care recipient’s BPSD (β = 0.17; *p* < 0.001) were associated with higher depressive symptoms over time.

Akaike’s Information Criteria (AIC) revealed that the inclusion of insomnia symptoms significantly improved the model fit when comparing it with a model that only contained the covariates (χ^2^(13) = 2289.67–2257.58 = 32.09; *p* < 0.01). Introducing insomnia symptoms in the model explained an additional 7.47% of the variance in depressive symptoms, and the final model explained 57.93% of the between-person variance.

### 3.3. Model 2: Longitudinal Relationship Between Depressive and Insomnia Symptoms

The results of the linear mixed model conducted to analyze the bidirectional relationship between depressive and insomnia symptoms are shown in Table 3. The coefficient for “time” was not significant, indicating that caregivers showed no significant changes in insomnia symptoms over time. The results showed that the time-varying value for depressive symptoms over time also indicated more insomnia symptoms over time (β = 0.17; *p* < 0.001; Model 2). This association remained significant after controlling for covariates. Additionally, being a non-smoker (β = −1.99; *p* < 0.05) and taking a higher number of medications (β = 1.10; *p* < 0.05) were associated with more insomnia symptoms over time.

Akaike’s Information Criteria (AIC) revealed that the inclusion of depressive symptoms significantly improved the model fit when comparing it with a model that only contained the covariates (χ^2^(13) = 2002.33–1984.60 = 17.73; *p* < 0.001). Introducing depressive symptoms into the model explained an additional 7.28% of the variance in insomnia symptoms, and the final model explained 25.74% of the between-person variance.

### 3.4. Secondary Analyses: Depression and Insomnia Symptoms and Time Since Being a Caregiver

Secondary analyses aimed to explore whether time spent as a caregiver was associated with depression and insomnia symptoms. Spearman correlation coefficient did not show statistically significant associations between months caregiving and depression (rs = 0.04; *p* = 0.60) or insomnia symptoms (rs = 0.06; *p* = 0.41). Also, the months of caregiving variable at baseline was categorized into three subgroups: (1) caregivers who had been performing this role between 3–12 months (n = 22); (2) caregivers who had been performing this role between 13–60 months (n = 89); and (3) caregivers who had been performing this role for 61 months or more (n = 44). One-way ANOVAs did not show statistically significant differences between these subgroups for depression (F = 0.12; *p* = 0.89) or insomnia symptoms (F = 0.19; *p* = 0.83).

## 4. Discussion

The objective of this study was to analyze the bidirectional association between insomnia and depressive symptoms with a longitudinal approach in a sample of family dementia caregivers who were interviewed during a two-year period.

The results showed that 37.4% of the participants reported clinically significant depressive symptoms at baseline, consistent with previous studies (Collins & Kishita, 2020), where 31.2% of caregivers were found to have depression. Regarding insomnia symptoms, the results showed that 27.5% of caregivers showed clinically significant insomnia symptoms at baseline. This value is smaller than previous findings (Peng & Chang, 2013), where approximately 50–74% of caregivers reported clinically significant sleep problems.

Our results were consistent with our research hypotheses and previous studies in non-caregiving populations (Alvaro et al., 2013; Franzen & Buysse, 2008; Sivertsen et al., 2012). Regarding our Hypothesis 1, insomnia symptoms were longitudinally associated with caregivers’ depressive symptoms. This association remained statistically significant after controlling for sociodemographic, health behaviors and medical factors, and caregiving stressors. The final model explained 57.93% of the between-person variance in caregivers’ depressive symptoms. Regarding our Hypothesis 2, depressive symptoms were longitudinally associated with caregivers’ insomnia symptoms, and this association remained statistically significant after controlling for the previously mentioned covariates. The final model explained 25.74% of the between-person variance in caregivers’ insomnia symptoms.

It was of special interest to analyze which covariates entered in Model 1 and Model 2 reached statistical significance in our study. In Model 1, multiple variables were statistically associated with depressive symptoms. However, in Model 2, care recipients’ BPSDs did not demonstrate a significant association with insomnia symptoms, even though the available literature consistently identifies them as the main source of sleep problems (Leggett et al., 2020) in family dementia caregivers. Thus, some further consideration must be given on this matter. One possible explanation can be drawn from the assessment scale. The RMBPC (Teri et al., 1992) only has one item on care-recipients’ nighttime behaviors (“He/she wakes you up at night”). Also, even though the RMBPC is a 24-item scale validated for Spanish family dementia caregivers (Nogales-González et al., 2015), the specific item regarding nighttime behaviors has not been validated for single use, nor has it been used in any previous research, as far as we are aware. It is possible that covering more specific sleep-related behaviors such as nocturnal wandering or falls could help to further explain the association between care recipients’ BPSDs and caregivers’ insomnia symptoms (Leggett et al., 2018). Another feasible explanation may be related to the statistical analysis carried out. In linear mixed models, a specific independent variable may have a bigger explanatory effect on the dependent variables. Thus, when including it in the model, the residual error is reduced and the other variables lose statistical relevance (Schupbach & Sprenger, 2011). Thus, when one independent variable explains a large portion of the variation in the dependent variable, it can absorb the explanatory power that might be otherwise attributed to other variables (West et al., 2022). In our model, it seems that the variable “depressive symptoms” has the biggest explanatory effect, and thus, other variables lose statistical significance. This has clinical implications, since our results suggest that targeting depressive symptoms in family dementia caregivers should be a primary focus in clinical practice for caregivers with insomnia.

These results add further empirical evidence to the notion that both insomnia and depression may operate as a risk factor for the other disorder (Sivertsen et al., 2012), making it possible to extend these findings to the caregiving population. The longitudinal approach helps provide a clearer picture of the interplay between symptoms of insomnia and depression (Peng & Chang, 2013). Additionally, our study also considered a broad range of important potential covariates, following recommendations by previous authors (Alvaro et al., 2013). Regarding these covariates, our results also support previous research that suggested that depression or insomnia symptoms may persist even after caregiving cessation. For example, the study by Robinson-Whelen et al. (2001) found that former caregivers’ depressive symptoms persisted up to three years after caregiving had ended. Another study by Rafnsson et al. (2017) even found that caregiving exit was related to increased depressive symptoms both in spousal and adult children caregivers. Similarly, the study by Gibson et al. (2023) outlined that many former caregivers reported persisting sleep problems associated with poor sleep habits established while caring. In our analysis, the variable “caregiving cessation” was not statistically associated with either depression or insomnia symptoms over time, adding further support to the idea that the stress derived from the caregiving situation may have long-lasting effects on caregivers’ mental health. On the other hand, secondary analyses also suggested that, once assuming the caregiving role, depression and insomnia symptoms seem to be rather stable over time. These results are in line with previous research by Rafnsson et al. (2017), who also found that entry into caregiving was associated with depressive symptoms and lower quality of life. In their study, those authors also did not find statistically significant differences in depressive symptoms between new caregivers and caregivers who had been assuming this role for a longer time. Thus, our study is consistent with the existing literature, extending this notion also to insomnia symptoms in family dementia caregivers.

This study has some limitations that need to be mentioned. First, the sample consisted of 155 voluntary caregivers, and so the sample may not be representative of the caregiving population at large. Secondly, despite the longitudinal design of the study, its results should be interpreted with caution. Future longitudinal studies with larger samples are needed to confirm the results. Other methodologies such as time-lagged studies or randomized controlled intervention studies could also be helpful to investigate causal associations. Additionally, this study used a self-report sleep measure instead of objective methods such as actigraphy. Also, data were not collected regarding previous clinical history of psychopathology or insomnia prior to assuming the caregiving role. Given that previous depression or insomnia are among the strongest predictors of recurrent episodes (Vitaliano et al., 1995), future studies should consider assessing participants’ clinical histories. We also did not collect information regarding over-the-counter medications for mood or insomnia (e.g., melatonin). Finally, the study sample consisted of participants both with and without clinically relevant depressive symptoms and/or insomnia symptoms. Although not addressed in this paper, it is worth noting here that 59.5% of caregivers with clinically significant depressive symptoms also showed clinically significant sleep problems at baseline. Likewise, 49.3% of caregivers with clinically significant insomnia symptoms also showed clinically significant depressive symptoms at baseline. Thus, it would be of great interest in future studies to consider caregivers without clinically relevant depressive symptoms or sleep problems at baseline and investigate which factors are associated with the development of a clinically relevant symptomatology over the study period.

Despite these limitations, this study is, to the best of our knowledge, the first to analyze the bidirectional relationship between depressive and insomnia symptoms in the dementia caregiving population using a longitudinal approach. Given that caregivers face a wide range of stressors (Vitaliano et al., 2004) that may have consequences for their mental and physical health (Schulz et al., 2020), these findings have potential implications for public health policies and clinical interventions with the caregiving population. According to a review study by Freeman et al. (2020), clinical trials of cognitive behavioral treatment for insomnia that include a depression outcome indicate that evidence-based treatments for insomnia also lead to reductions in depression. Focusing on the caregiving population, sleep quality seems to improve in caregivers who receive behavioral interventions (Gao et al., 2019). Thus, interventions targeting depressive symptoms might improve their efficacy if they also treat insomnia symptoms.

## Figures and Tables

**Table 1:** Characteristics of the sample at baseline and yearly assessments.

Variable	Baseline (n = 155)	Year 1 (n = 139)	Year 2 (n = 123)
	Mean (SD) or Percentage (n)/Scale Range	Mean (SD) or Percentage (n)/Scale Range	Mean (SD) or Percentage (n)/Scale Range
Women, %	64.50 (100)	65.00 (91)	68.03 (83)
Age	62.26 (12.22)/32–87	62.70 (11.86)/33–88	63.29 (12.21)/34–89
Months caregiving	51.45 (35.96)/3–180	63.28 (35.47)/15–192	77.48 (37.73)/27–204
Daily hours of caregiving	13.18 (8.36)/1–24	12.69 (8.87)/0–24	11.08 (9.11)/0–35
Caregiving cessation, %	0 (0)	20.00 (31)	29.70 (46)
Health problems	1.96 (1.49)/0–7	1.72 (1.47)/0–7	1.83 (1.54)/0–8
Hours of exercise per week	3.23 (4.12)/0–22	3.98 (5.16)/0–35	3.66 (4.37)/0–18
Leisure activities	6.30 (2.77)/0–12	5.99 (2.83/0–12	5.89 (2.80)/0–12
Alcohol intake	2.97 (4.73)/0–30	2.86 (3.85)/0–20	3.07 (4.81)/0–24
Smoker, %	21.30 (33)	17.40 (27)	14.20 (22)
Medication intake	0.34 (0.64)/0–3	0.36 (0.81)/0–5	0.28 (0.52)/0–2
Behavioral and Psychological Symptoms of Dementias	17.57 (14.44)/0–68	16.39 (13.80)/0–63	14.71 (14.31)/0–53
Depressive symptoms	15.01 (10.18)/0–44	14.62 (10.53)/0–45	14.38 (9.70)/0–41
Caregivers with clinically relevant depressive symptoms, %	37.4 (58)	37.0 (51)	38.2 (47)
Insomnia symptoms	6.10 (5.53)/0–25	6.76 (5.57)/0–23	6.33 (5.37) 0/21
Caregivers with clinically relevant insomnia symptoms, %	27.5 (42)	25.0 (34)	29.3 (36)

*Note*. values for dichotomic variables are: gender (0 = male; 1 = female), caregiving cessation (0 = still the main caregiver; 1 = no longer the main caregiver), smoker (0 = nonsmoker; 1 = smoker). Caregivers with clinically relevant depressive symptoms: CES-D scores ≥ 16. Caregivers with clinically relevant insomnia symptoms: ISI scores ≥ 10.

**Table 2:** Model 1. Linear mixed-effects model for depressive symptoms over time.

Variable	Estimate	SE	Df	t	*p*	95% Confidence Interval	
						Lower Bound	Upper Bound
Intercept	23.69	3.60	175.98	6.58	<0.001	16.59	30.80
Time	−0.49	0.45	218.22	−1.10	0.27	−1.38	0.39
Female gender	−0.38	1.21	148.48	−0.32	0.75	−2.78	2.01
Age at baseline	−0.15	0.05	172.84	−2.82	<0.01	−0.25	−0.04
Months caregiving	−0.01	0.01	129.48	−0.36	0.72	−0.03	0.02
Daily hours caregiving	−0.03	0.05	315.77	−0.61	0.54	−0.14	0.07
Caregiving cessation	0.00	1.44	277.75	0.00	0.10	−2.84	2.83
Health problems	0.27	0.33	306.38	0.80	0.42	−0.38	0.92
Hours of exercise per week	−0.03	0.07	287.62	−0.43	0.66	−0.16	0.10
Leisure activities	−0.94	0.17	316.38	−5.61	<0.001	−1.26	−0.61
Alcohol intake per month	−0.04	0.11	297.70	−0.39	0.699	−0.26	0.18
Smoking status	2.39	1.24	264.46	1.93	0.06	−0.05	4.83
Medication intake	2.31	0.72	300.12	3.22	<0.01	0.90	3.72
Behavioral and psychological symptoms	0.17	0.03	304.60	5.16	<0.001	0.11	0.24
Insomnia symptoms	0.41	0.08	313.37	4.91	<0.001	0.25	0.57

Data are given as slopes (B). Intercepts correspond to depressive symptoms for male’s caregivers at baseline. Gender is coded as 0 = male or 1 = female. Caregiving cessation is coded as 0 = still caregiver or 1 = no longer caregiver, and time is entered as a linear variable with 0 = baseline scores, 1 = 1 year follow-up, and 2 = 2 years follow-up from baseline.

**Table 3:** Model 2. Linear mixed-effects model for insomnia symptoms over time.

Variable	Estimate	SE	Df	t	*p*	95% Confidence Interval	
						Lower Bound	Upper Bound
Intercept	7.17	2.45	182.66	2.92	<0.01	2.33	12.01
Time	0.10	0.28	214.79	0.35	0.73	−0.46	0.66
Female gender	0.92	0.82	151.68	1.11	0.27	−0.71	2.54
Age at baseline	−0.02	0.04	180.23	−0.50	0.62	−0.09	0.05
Months caregiving	0.01	0.01	133.36	1.36	0.18	−0.01	0.03
Daily hours caregiving	0.02	0.03	308.91	0.63	0.53	−0.05	0.09
Caregiving cessation	0.71	0.92	262.76	0.78	0.43	−1.09	2.52
Health problems	0.39	0.21	313.06	1.81	0.07	−0.03	0.81
Hours of exercise per week	−0.05	0.04	273.87	−1.26	0.21	−0.14	0.03
Leisure activities	−0.20	0.11	315.67	−1.78	0.07	−0.42	0.02
Alcohol intake per month	0.02	0.07	309.36	0.26	0.79	−0.12	0.16
Smoking status	−1.77	0.82	285.42	−2.16	<0.05	−3.38	−0.16
Medication intake	1.10	0.47	308.83	2.32	<0.05	0.17	2.03
Behavioral and psychological symptoms	−0.01	0.02	316.80	−0.60	0.55	−0.06	0.03
Depressive symptoms	0.17	0.04	315.25	4.83	<0.001	0.10	0.24

Data are given as slopes (B). Intercepts correspond to depressive symptoms for male’s caregivers at baseline. Gender is coded as 0 = male or 1 = female. Caregiving cessation is coded as 0 = still caregiver or 1 = no longer caregiver, and time is entered as a linear variable with 0 = baseline scores, 1 = 1 year follow-up, and 2 = 2 years follow-up from baseline.

## Data Availability

Study was not preregistered. The data presented in this study are available on request from the corresponding author due to the ongoing status of the project.
